# Digital Workflow for the Design, Manufacture, and Application of Custom-Made Short Implants With Wing Retention Device

**DOI:** 10.3389/fbioe.2022.885746

**Published:** 2022-06-08

**Authors:** Zexian Xu, Zhen Yang, Jianjun Yang

**Affiliations:** ^1^ Department of Oral and Maxillofacial Surgery, The Affiliated Hospital of Qingdao University, Qingdao, China; ^2^ School of Stomatology of Qingdao University, Qingdao, China; ^3^ Dental Digital Medicine and 3D Printing Engineering Laboratory of Qingdao, Qingdao, China; ^4^ Department of Prosthodontics, Peking University School and Hospital of Stomatology, Beijing, China; ^5^ National Clinical Research Center for Oral Diseases, Beijing, China; ^6^ National Engineering Laboratory for Digital and Material Technology of Stomatology, Beijing, China; ^7^ Beijing Key Laboratory of Digital Stomatology, Beijing, China

**Keywords:** bone height, custom-made, CAD/CAM, dental implant, digital workflow

## Abstract

The severe deficiency of vertical bone height in the posterior maxillary region poses a challenge to implant restoration. In response to this issue, this article introduces a custom-made short implant with a wing retention structure and describes a precise and minimally invasive dental implant restoration scheme with digital technology.

## Introduction

Dental implant restoration performed in the loss of posterior maxillary teeth can be a challenge owing to the insufficient vertical bone volume resulting from the resorption of an alveolar bone and the pneumatization of a maxillary sinus (Mu et al., 2020). It has been found that the residual bone height (RBH) of some patients is only 1–2 mm, posing difficulties in stabilizing the implant initially (Pignaton et al., 2019). When RBH is 4 mm or less, the lateral sinus floor elevation with delayed implant placement is suggested in clinical use. However, it usually requires bone grafting, and prolongs the recovery time of patients with missing teeth. Furthermore, lateral antrostomy surgery has a high risk and may damage the branches of the posterior alveolar artery. In addition, the surgery process may cause tearing and perforation of the medial wall mucosa, which in turn affects osteogenesis (Gonzalez et al., 2014; Rammelsberg et al., 2020). Considering this fact, Bedrossian et al. reported a zygomatic implant with a length of 30–52.5 mm placed through a sinus cavity and fixed in a zygomatic bone to guarantee the initial stabilization (Bedrossian et al., 2002). However, this technique is complex and invasive, often associated with serious complications like infection, bleeding, and nerve injury (Agliardi et al., 2021).

With the digitization of modern medicine, some scholars are proposing the application of subperiosteal implants for patients with severe bone insufficiency in the posterior maxillary region (Gellrich et al., 2017a; Jehn et al., 2020). Gellrich et al. designed an improved subperiosteal implant (Individual Patient Solution-dental, IPS-d), equipped with dozens of screw holes. The IPS-d was made of type III titanium (Ti6Al4V) as material and printed by selective laser melting technology, and fixed by miniature screws to complete the denture restoration in the missing area. These have promoted the idea and development of an innovative skeletal anchorage system for prosthodontic rehabilitation. However, in order to ensure the stability of the implant, the titanium plate is designed with a large spread area. In addition, the “pile” structure is integrated with the titanium plate, without a platform transfer structure, so it is difficult to obtain a good biological seal of gingival soft tissue. The clinical application of zygomatic implant and improved subperiosteal implant in the restoration of missing teeth with severe bone insufficiency is also limited by the disadvantages of large surgical trauma, many postoperative complications, and unguaranteed long-term restoration effect. Till now, treating patients with the RBH seriously deficient (RBH<3 mm) in the posterior maxillary area remains difficult.

Therefore, a custom-made short implant with a wing retention structure has been developed with digital technology. This new implant is expected to provide long-term stability through the retention of a wing device, with no need for maxillary sinus lateral antrostomy. Moreover, the surgery process is relatively safe, and can reduce the trauma, pain, and complications of patients.

## Methods

The following method was used to design, fabricate, and implant a new implant.

**FIGURE 1:**
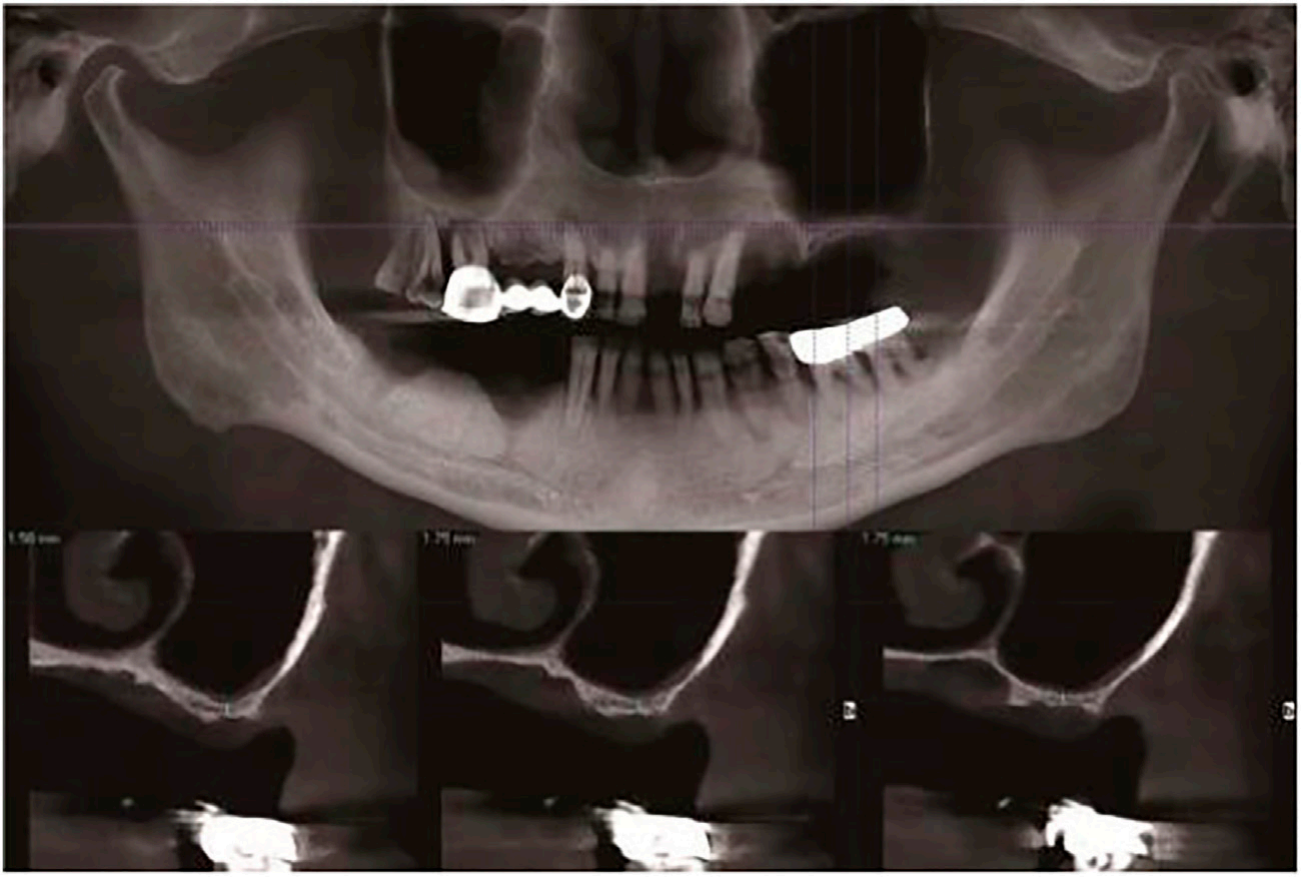
Measurements of the CBCT for preoperative evaluation.

**FIGURE 2:**
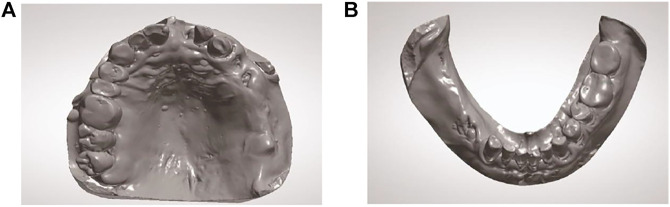
Intraoral scans of the patient’s arches. **(A)** Maxillary STL occlusal view. **(B)** Mandibular STL occlusal view.

**FIGURE 3:**
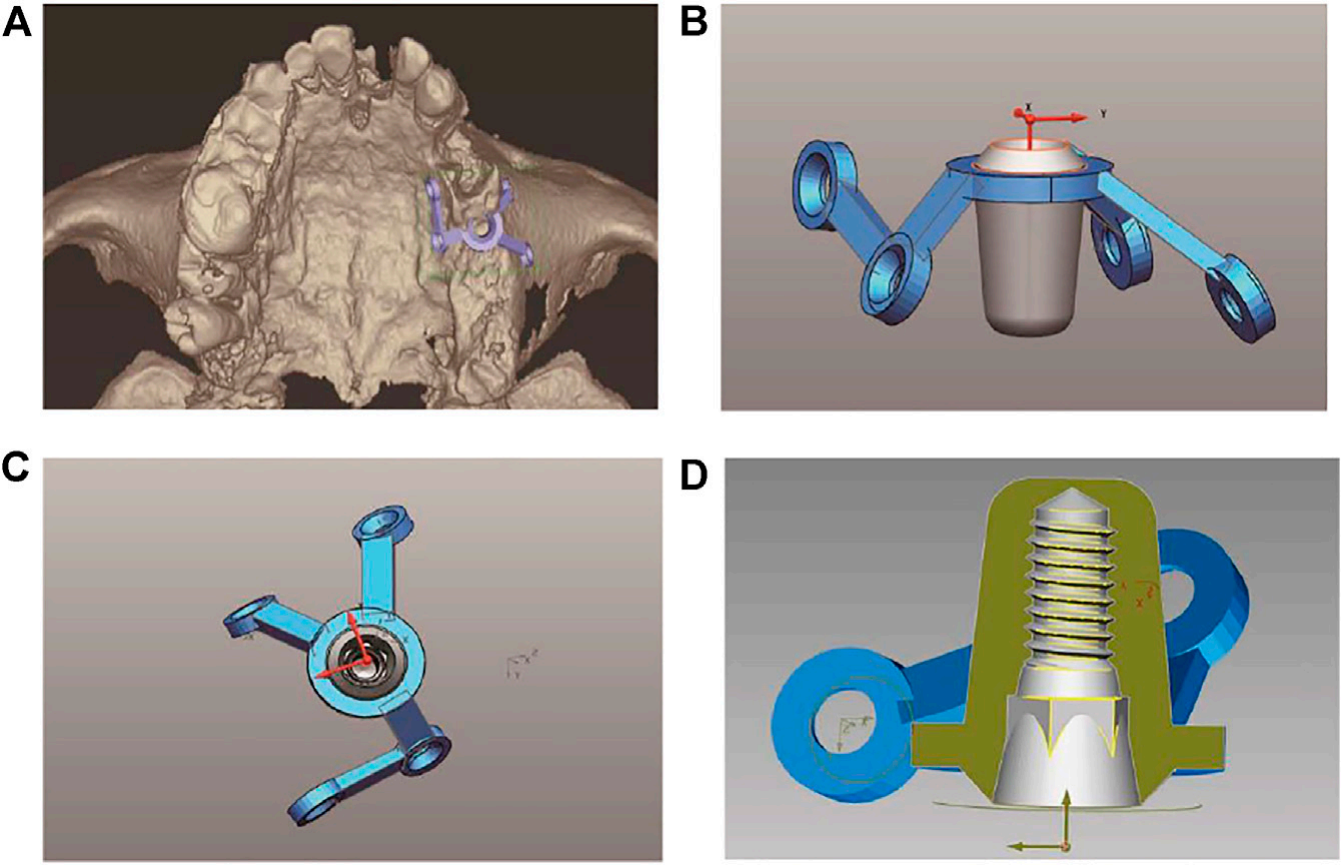
Presurgical design. **(A)** The virtual model of the custom-made implant and its position in the 3D modeling of the alveolar bone. **(B)** The side view of the virtual model of the custom-made implant. **(C)** The top view of the virtual model of the custom-made implant. **(D)** The internal view of the virtual model of the custom-made implant.

**FIGURE 4:**
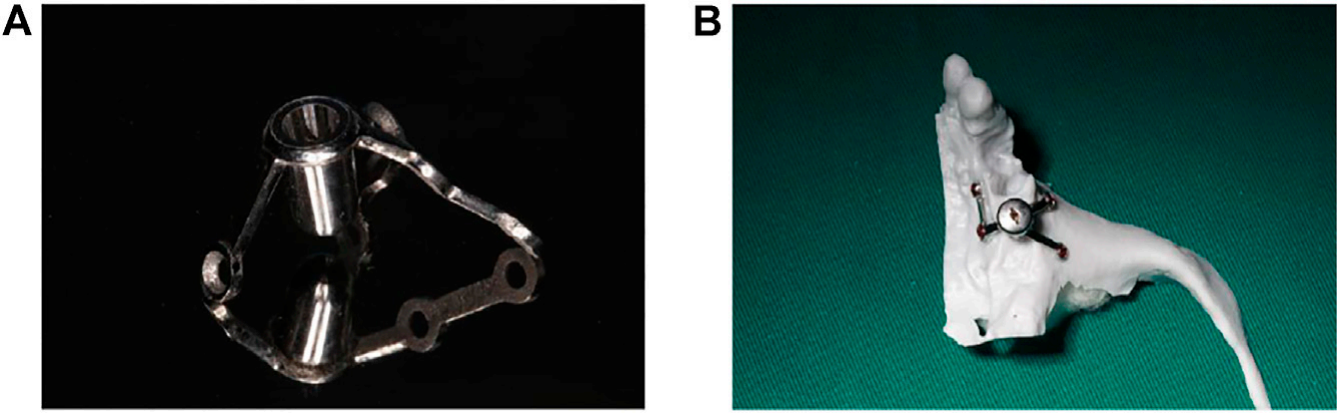
The custom-made implant and 3D printed model of the bone. **(A)** The custom-made implant. **(B)** The custom-made implant on a 3D bone reconstruction resin model.

**FIGURE 5:**
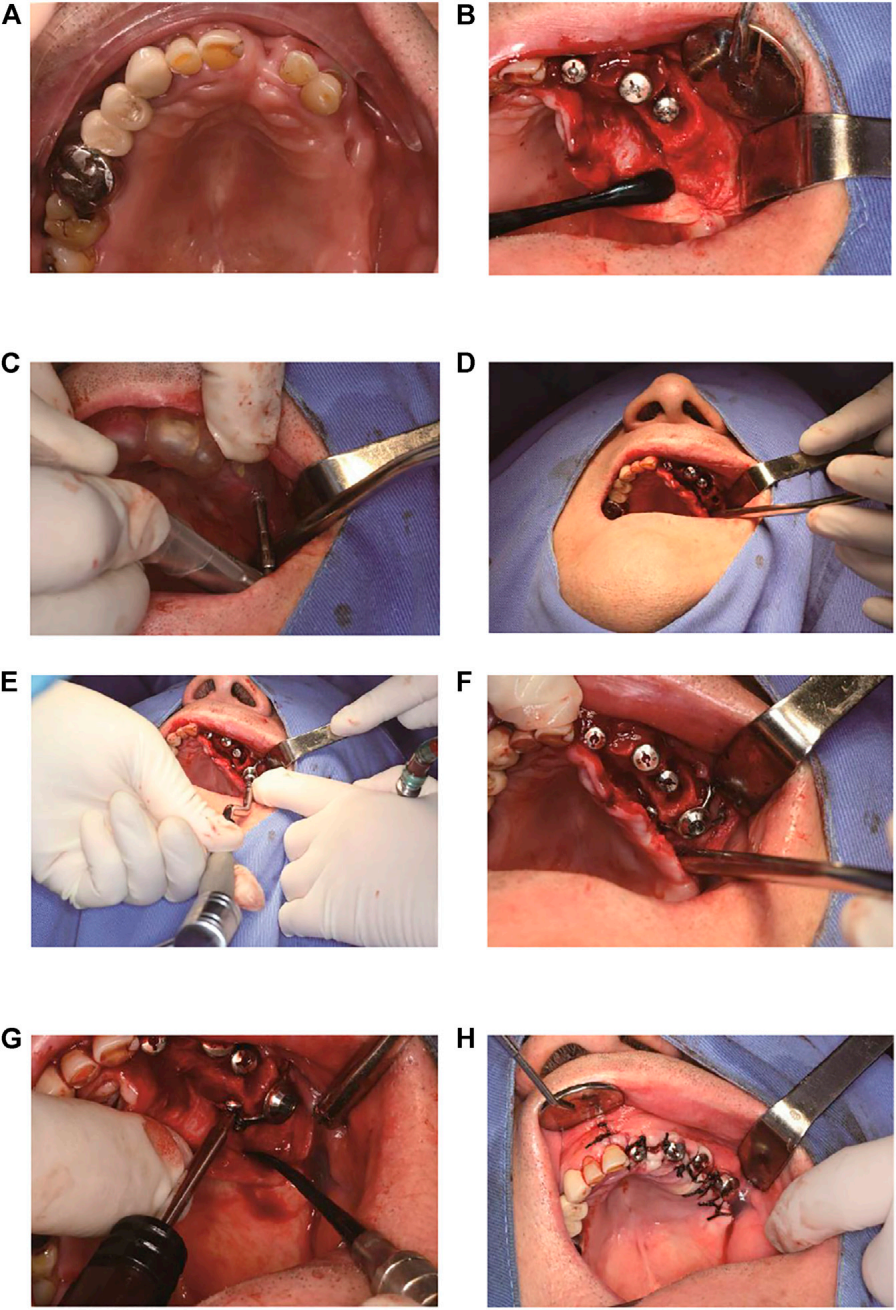
The brief surgical procedure. **(A)** Preoperative intraoral view. **(B)** Anatomy of the residual bone. **(C)** Location of the implant by implant guide template. **(D)** The confirmation of implant socket direction and depth. **(E)** Placement of the implant. **(F)** The implant is in position. **(G)** Fixation with several mini-screws. **(H)** Sutures.

**FIGURE 6:**
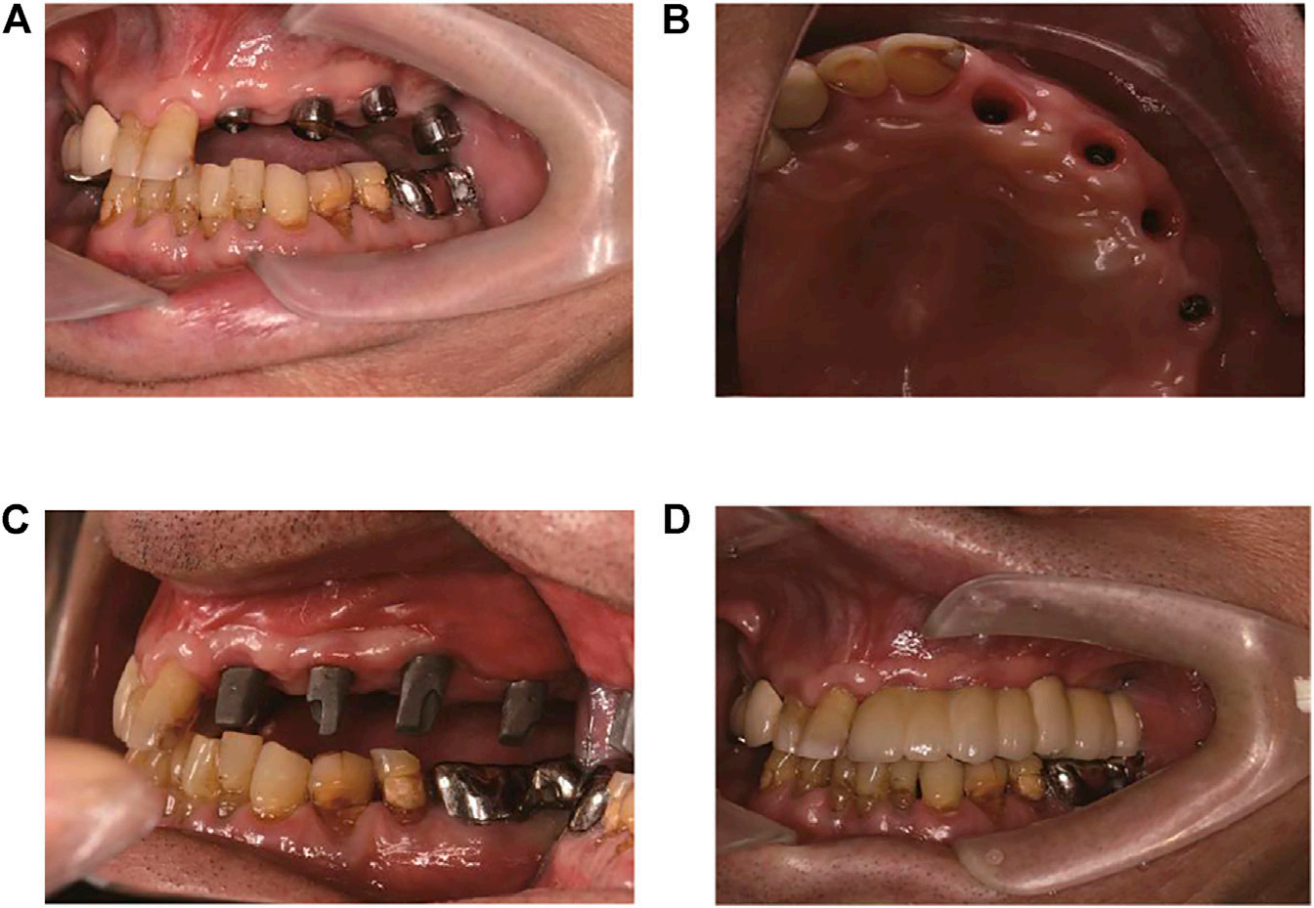
The prosthodontics. **(A)** 6-month postoperative view. **(B)** Soft tissue cuffs appearance. **(C)** Custom-made abutments in place. **(D)** Application of the final prosthetic denture.

**FIGURE 7:**
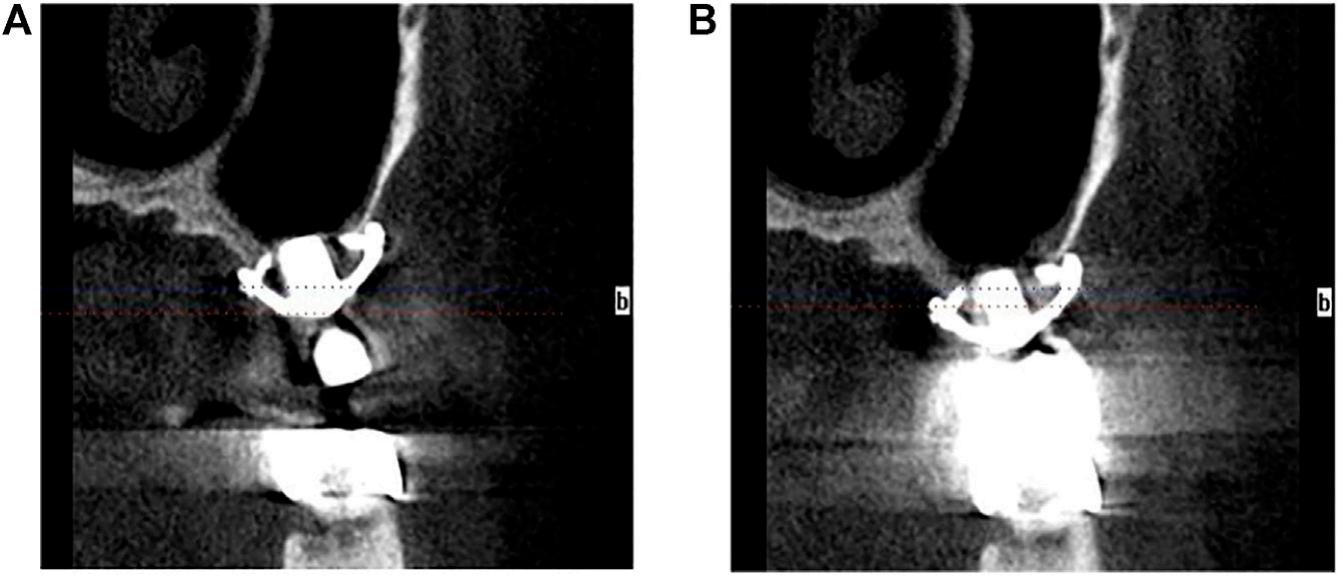
The CBCT images. **(A)** 6-month postoperative CBCT image. **(B)** 12-month postoperative CBCT image.

1) Make a preoperative cone-beam computed tomography (CBCT, KaVo, Germany) scan of a patient to obtain high-resolution digital imaging and communications in medicine (DICOM) data for diagnosis and treatment planning (Figure 1). The scanning conditions of CBCT are as follows: the tube voltage is 120 kV, the tube current is 5 mA, the exposure time is 27 s, and the scanning layer thickness is 250 μm. Patients with a residual bone height (RBH<3 mm) of less than 3 mm in the posterior maxillary region were selected.
2) Use a model scanner (D2000; 3Shape) to scan the maxillary and mandibular plaster casts, and export the model data in a standard tessellation language (STL) file format (Figures 2A,B).
3) Register CBCT data with model scanning data to obtain a virtual 3D bone reconstruction model of the patient. Design the position and shape of the custom-made implant by 3Shape dental system (3Shape, Denmark) according to the model (Figure 3A). Design the body part of the implant in the shape of a conical structure that can be inserted into the implant hole. Make sure that the lower surface of the wing retention structure closely fits the outer surface of the alveolar bone (Figure 3B). Design the upper part of the short implant as an implant platform shaped as a frustum of a cone with a slope of 45° and height of 1 mm (Figure 3C). Use an inverted round table hole with a morse taper, an inner hexagonal hole, and a threaded hole to fix the abutment (Figure 3D). Design screw hole locations to avoid damaging important anatomical structures and healthy adjacent teeth.
4) Transfer the virtual plan to a manufacturer and fabricate the custom-made implant *via* a seven-axis lathe (480MT; Willemin) (Figure 4A). Design and manufacture a resin surgical template to facilitate implant placement. Test the accuracy and fitness of the custom-made implant on a 3D bone reconstruction resin model (Figure 4B).
5) Carry out periodontal scaling and oral hygiene education for patients before the surgery. Disinfect the oral cavity with iodophor and achieve local infiltration anesthesia with articaine. Make a buccal vertical incision (>5 mm) at the middle of the adjacent tooth, beneath the mucogingival junction. Then make a crestal incision to disclose the alveolar crest and elevate a full-thickness mucoperiosteal flap on the buccal side (Figures 5A,B) (Liu et al., 2021). Prepare an alveolar socket with the implant guide template (Figures 5C,D), and then place the custom-made implant with the healing abutment by hammering the concave osteotome (Dentium, South Korea) (Figures 5E,F). Fix the wings of the retention structure with several self-tapping miniature screws (Shuangyang, China) in the correct position (Figure 5G). Close the flaps with interrupted by using 3-0 Mersilk sutures. (Figure 5H).
6) Remove the healing abutments 6 months after the surgery (Figures 6A,B), and then do the final restoration (Figures 6C,D). Make an impression with polyether silicone rubber impression material and use a zirconia ceramic crown for restoration. Restoration is screw-retained rather than cemented connection of the abutment to the implant body (Liu et al., 2019).
7) Make 6-month and 12-month postoperative CBCT scans to assess the custom-made implant (Figures 7A,B) and conduct a follow-up.

## Results and Discussion

The patients with severe bone deficiency in the posterior maxillary region successfully completed the placement of custom-made short implants according to this method. In this study, a new custom-made implant is proposed, which consists of two parts: a wing retention structure and a short implant. The wing retention is similar to the subperiosteal retention and is fixed by tiny screws, which is the main fixation of the custom-made implant. However, it is different from a conventional subperiosteal implant regarding the design process. A conventional subperiosteal implant is fixed with titanium plates. In order to ensure stability, the titanium plates need to cover a large area of the bone surface, and a large flap area is required during surgery, which easily affects the blood supply to soft tissue and causes ischemic necrosis in the mucosal area, leading to the failure of implantation. At the same time, the “post” on the upper part of the titanium plates is used to support the denture for restoration. As there is no platform transfer structure, the post structure makes it impossible to attach the gingival soft tissue to the implant to form a biological seal (Mangano et al., 2020). The defective design makes the prepared subperiosteal implant less resistant to bacterial infection, thus affecting the long-term therapeutic effect. On the contrary, this custom-made implant can be implanted at a single tooth missing position. The coverage area of the wing retention is controllable and only needs to be flipped routinely. The wing retention design follows the principles of the triangular retainer and multi-direction placement to ensure mutual antagonism of mechanics. In addition,, the wings are retained by miniature screws to ensure the initial and long-term stability of the implant. At the same time, the short implant structure is preserved, and its length is about 5 mm. The short implant is submerged downward and screw-retained at the bone level. The platform transfer structure is designed to ensure that good soft tissue closure can be formed around the implant after implantation, which is conducive to the long-term stability of the implant. The custom-made implant is neither a subperiosteal implant nor a traditional short one. It retains the short implant structure and adopts an ultra-new concept of wing plate retention. It is a good implant, especially for patients with severe bone insufficiency.

With the development of computer-aided design and computer-aided manufacturing (CAD/CAM), new treatments have been introduced into dentistry (Taha et al., 2021). By analyzing and exporting CBCT data, a 3D resin model of bone reconstruction and a piece of implant guide plate that could accurately locate the implantation position were printed. After that, with the help of CAD/CAM techniques, this study digitally cut a custom-made implant with high mechanical strength with an advanced seven-axis lathe. In this way, the custom-made implant has good biomechanics due to its integrated structure. Previous FEM-stress-test studies can confirm this view (Gellrich et al., 2017b). Moreover, the virtual design of the implant could be precisely transferred into the mouth, ensuring that the lower surface of the wing retention could be closely attached to the outer surface of the alveolar bone. This workflow realizes the digitalization of the implant scheme from preoperative design to precise intraoperative guidance, and finally to postoperative repair.

Compared with traditional implants, the custom-made implants are implanted without bone grafting, thus improving the implant precision, reducing the intraoperative time, and making the operation easier. Despite the underlying problem that it takes about 1 day to design and manufacture the implant, the patients who cannot undergo regular implants due to severe bone insufficiency have supported this technique and taken the implant restoration treatment up to date. Hence, the custom-made implant is more economical, practical, and safer, which is a different modality of treatment from maxillary sinus floor elevation, zygomatic implants, or subperiosteal implants.

## Conclusion

This article introduces a new invention of a short implant with a wing retention structure that can be fixed to both subperiosteum and alveolar bones, and describes a precise, minimally invasive, and effective dental implant restoration scheme custom-designed for patients with severe vertical bone height insufficiency in the posterior maxillary region.

## Data Availability

The original contributions presented in the study are included in the article/Supplementary Material, further inquiries can be directed to the corresponding author.
