# Supplementary figures and images for: Digital Workflow for the Design, Manufacture, and Application of Custom-Made Short Implants With Wing Retention Device
Source: Front Bioeng Biotechnol. 2022 Jun 8;10:885746. doi: 10.3389/fbioe.2022.885746 (PMC9213801; doi:10.3389/fbioe.2022.885746)

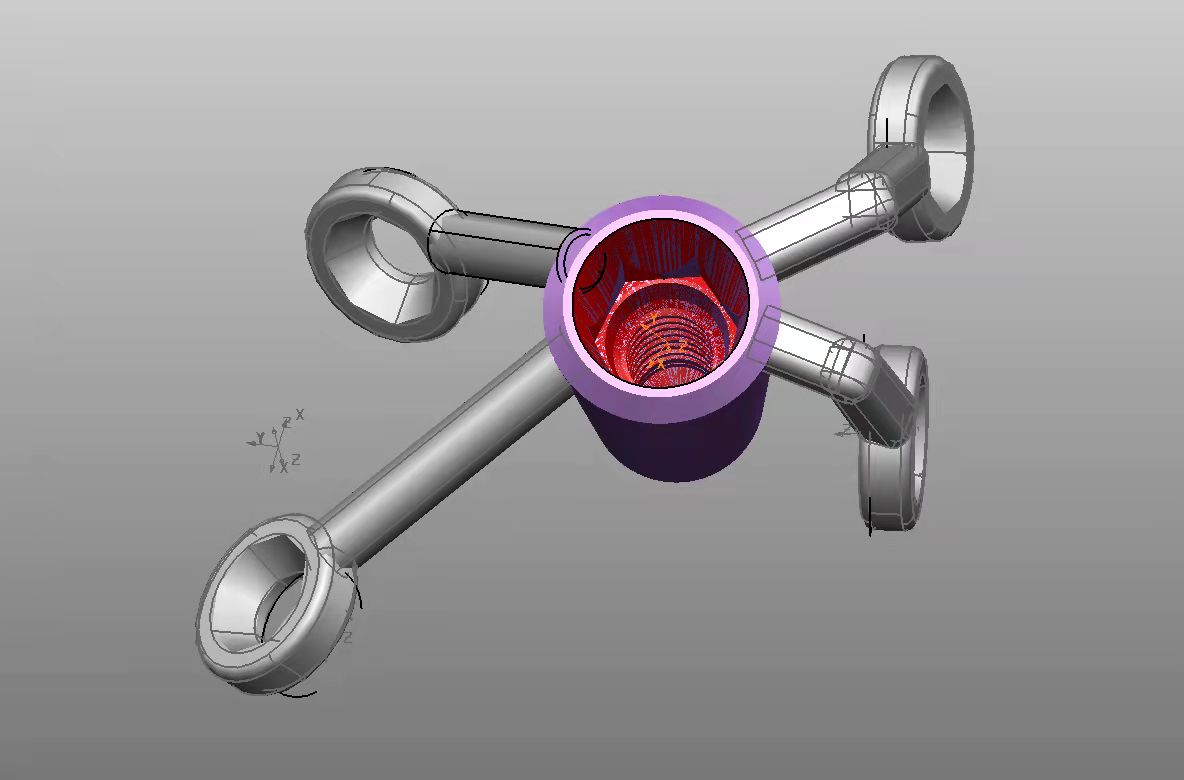

Supplement: Supplementary file 1 [file Image1.JPEG]
